# Gene Sequencing and Phylogenetic Analysis: Powerful Tools for an Improved Diagnosis of Fish Mycobacteriosis Caused by *Mycobacterium fortuitum* Group Members

**DOI:** 10.3390/microorganisms9040797

**Published:** 2021-04-10

**Authors:** Davide Mugetti, Mattia Tomasoni, Paolo Pastorino, Giuseppe Esposito, Vasco Menconi, Alessandro Dondo, Marino Prearo

**Affiliations:** 1 Istituto Zooprofilattico Sperimentale del Piemonte, Liguria e Valle d’Aosta, Via Bologna 148, 10154 Torino, Italy; mattia.tomasoni@izsto.it (M.T.); paolo.pastorino@izsto.it (P.P.); vasco.menconi@izsto.it (V.M.); alessandro.dondo@izsto.it (A.D.); marino.prearo@izsto.it (M.P.); 2 Dipartimento di Medicina Veterinaria, Università degli Studi di Sassari, Via Vienna 2, 07100 Sassari, Italy; gsesposito@uniss.it

**Keywords:** *Mycobacterium fortuitum*, *Mycobacterium peregrinum*, Mycobacteria Other Than Tuberculosis (MOTT), Nontubercolus mycobacteria (NTM), fish mycobacteriosis, zoonoses, heat shock protein, Sanger sequencing, phylogenetic tree

## Abstract

The *Mycobacterium fortuitum* group (MFG) consists of about 15 species of fast-growing nontuberculous mycobacteria (NTM). These globally distributed microorganisms can cause diseases in humans and animals, especially fish. The increase in the number of species belonging to MFG and the diagnostic techniques panel do not allow to clarify their real clinical significance. In this study, biomolecular techniques were adopted for species determination of 130 isolates derived from fish initially identified through biochemical tests as NTM belonging to MFG. Specifically, gene sequencing and phylogenetic analysis were used based on a fragment of the gene encoding the 65 KDa heat shock protein (*hsp65*). The analyzes made it possible to confirm that all the isolates belong to MFG, allowing to identify the strains at species level. Phylogenetic analysis substantially confirmed what was obtained by gene sequencing, except for six strains; this is probably due to the sequences present in NCBI database. Although the methodology used cannot represent a univocal identification system, this study has allowed us to evaluate its effectiveness as regards the species of MFG. Future studies will be necessary to apply these methods with other gene fragments and to clarify the real pathogenic significance of the individual species of this group of microorganisms.

## 1. Introduction

The *Mycobacterium fortuitum* group (MFG) includes several rapidly growing nontuberculous mycobacteria (NTM) with very similar cultural, biochemical, and molecular features. The group takes its name from the most representative species, *Mycobacterium fortuitum*, described for the first time in 1938 [1] and included in group IV (rapid growers mycobacteria) of the Runyon’s classification [2]. With the improvement of diagnostic techniques, this group has undergone several additions and taxonomic changes as happened for most species of the genus *Mycobacterium*. This is evident also in the founding species of MFG, divided into two subspecies, *M. fortuitum* subsp. *fortuitum* and *M. fortuitum* subsp. *acetamidolyticum* [3]. Based on biochemical and cultural characteristics, several authors had proposed a division of *M. fortuitum* isolates into three biovars [4], subsequently separated into two distinct species (*M. fortuitum* and *M. peregrinum*) and a third biovariant complex with two unnamed taxa [5]. However, Schinsky and collaborators [6] demonstrated that the aforementioned “third biovariant complex” was composed of different species, subsequently named *M. boenickei*, *M. brisbanense*, *M. houstonense*, and *M. neworleansense*. Based on Bergey’s Manual and papers of the International Journal of Systematic and Evolutionary Microbiology regarding the official nomenclature, the MFG currently includes 13 NTM species: *M. alvei*, *M. boenickei*, *M. brisbanense*, *M. conceptionense*, *M. farcinogenes*, *M. fortuitum* (subs. *fortuitum*/*acetamidolyticum*), *M. houstonense*, *M. neworleansense*, *M. peregrinum*, *M. porcinum*, *M. senegalense*, *M. septicum*, and *M. setense* [7,8,9,10,11,12,13]. Moreover, other species closely related to MFG are reported, although not officially included in the group: specifically, these mycobacteria are *M. arceuilense*, *M. lutetiense* and *M. montmartrense* [14].

The importance of MGT studying lies in that several of its members are implicated in human and animal diseases. Being ubiquitous bacteria, the infection sources are represented by both natural (water and soil) [15,16] and anthropic environments (water distribution systems, swimming pools) [17,18,19,20]. Concerning human medicine, mycobacteria belonging to MFG are mainly the cause of dermal infections in immunocompromised patients [21]. *Mycobacterium fortuitum* is recognized as an etiological agent of pulmonary [22], bone [23], skin and soft tissue diseases following surgery [24] and catheter-associated infections [25]. Albeit sporadically, cases of infections in human due to members of the MFG are also reported in literature for *M. alvei* [26], *M. brisbanense* [27], *M. conceptionense* [28], *M. houstonense* [29], *M. peregrinum* [30,31], *M. porcinum* [32], *M. senegalense* [33,34], and *M. septicum* [35]. 

As mentioned, other animal species are also susceptible to NTM infection belonging to the MFG. *M. fortuitum* has been found in several terrestrial animal species, including bovine with mastitis [36,37], wild boars [38], dogs [39], and cats [40]. *Mycobacterium senegalense* and *M. farcinogenes* are well known in Africa as bovine pathogens, in which they are causative agents of a cutaneous diseases called “farcy” [7,41]. These same mycobacteria caused abscesses and death in guinea pigs following experimental infection [42]. Similar to what has been described for humans, other members of MFG have also been reported sporadically for wild and domestic animals [8,43,44,45,46].

However, based on scientific literature, the highest reports number of MFG members infections are in fishes. *Mycobacterium fortuitum*, like *M. marinum* and *M. chelonae*, is recognized as the main causative agent of fish mycobacteriosis [47]. Usually, these pathologies begin with nonspecific clinical signs (emaciation, abnormal behavior, scale loss, ulcerative skin lesion, skeletal deformities), then can become chronic with the formation of whitish military nodules in parenchymatous organs (liver, spleen, kidney) [48]. Mycobacteriosis caused by this group of pathogens affects both fresh and saltwater fishes, especially with regard to ornamental species [49,50,51]. Despite the large number of studies, MFG members isolated from fish are not always identified at the species level. In fact, it is possible to find works in which is not possible to discriminate between closely related species or the isolates are indicated as mycobacteria belonging to the MFG [52,53]. This is particularly evident as regards the isolates recognized with the sole use of biochemical tests, which do not allow to discriminate between the various species of MFG (Table 1). 

Therefore, it is likely that over the years there has been an underestimation of the less known and more recent *Mycobacterium* species of belonging to MFG. In addition, causing a loss of information, this lack of identification does not allow to attribute an accurate clinical significance to the different species of MFG. Thus, the aim of this study is to identify at species level the mycobacteria isolated from fish previously recognized by biochemical tests as *M. fortuitum* or as members belonging to the MFG. 

## 2. Materials and Methods

### 2.1. Strains Collection and Preliminary Identification

To perform this study, strains of NTM isolated from fish identified by biochemical tests as MFG members were selected during the years 2015–2019. The strains were isolated from parenchymatous organs (spleen, liver, kidney) of fish with external clinical signs attributable to mycobacteriosis. Before inoculation on Löwenstein–Jensen (Microbiol, Uta-Cagliari, Italy) and Stonebrink medium (Microbiol, Uta-Cagliari, Italy), the organs were homogenized, washed with a 1.5% aqueous solution of cetylpyridinium chloride monohydrate (AppliChem, Darmstadt, Germany) for 30 min and centrifuged for 20 min to obtain a pellet. For the first isolation, two tubes for each medium were incubated at 28 ± 1 °C and at 37 ± 1 °C for two months, respectively. All tubes were constantly checked for evaluating microorganisms growth. At the end of the incubation, colonies grown were then subjected to Ziehl-Neelsen (ZN) staining and biochemical tests as described by Kent and Kubica [55] with updates of Magee and Ward [54]. The tests carried out are those listed previously in Table 1. Following preliminary identification, the strains were stored in cryobank at −80 °C pending subsequent analyses. 

### 2.2. DNA Extraction and Amplification

The stored strains were reactivated using Middlebrook 7H9 broth (Microbiol, Uta-Cagliari, Italy) incubated at 28 ± 1 °C. Then, the broth was inoculated by a 10 μL sterile disposable loop in Löwenstein–Jensen medium to allow a better visualization of the colonies; this medium was incubated again at 28 ± 1 °C. Colonies were placed in a sterile eppendorf containing 200 μL of nuclease-free water (Sigma-Aldrich, St. Louis, MO, USA) and the bacterial DNA was extracted by ExtractMe Genomic DNA kit (Blirt S.A., Gdańsk Poland) following the manufacturer’s guidelines. The extracted nucleic acid was immediately tested or stored at −20 °C before amplification. 

For species identification, the protocol proposed by Telenti et al. [56] for the amplification of a 441 bp fragment of the 65 kDa heat shock protein (*hsp65*) gene was chosen. Compared to the original protocol, the PCRs were conducted in a volume of 50 μL using 25 μL of PrimeDirect™ Probe RT-qPCR Mix, with UNG (Takara Bio Inc., Shiga, Japan), primers Tb11 (5′-ACCAACGATGGTGTGTCCAT) and Tb12 (5′-CTTGTCGAACCGC-ATACCCT) at the concentration of 10 μM, 5 μL of template and nuclease-free water (Sigma-Aldrich, St. Louis, MO, USA) to bring to volume. A reference strain of *M. fortuitum* (*M. fortuitum* subsp. *fortuitum* da Costa Cruz, ATCC^®^ 6841™) was used as PCR positive control and water as negative control. PCR were performed on a 2720 Thermal Cycler (Applied Biosystems, Waltham, MA, USA) using the following thermal protocol: 50 °C for 2 min for Uracil-DNA Glycosylase (UDG) activation, 96 °C for 2 min for the initial denaturation, 40 amplification cycles (denaturation: 95 °C × 30′; annealing: 59 °C × 30′; extension: 72 °C × 30′) and 72 °C for 5 min for the final elongation.

PCR products were visualized using 2% agarose gel (Merck, Darmstadt, Germany), prepared using tris acetate-EDTA buffer 1× (Merck Millipore, Darmstadt, Germany) and GelRed^®^ Nucleic Acid Gel Stain (Biotium, Fremont, CA, USA). Fragment size was assessed using AmpliSize molecular ruler 50–2000 bp ladder (Bio-Rad, Segrate, Italy).

### 2.3. Sanger Sequencing and Species Determination

PCR products were purified by columns using Extractme DNA Gel-Out kit (Blirt S.A., Gdańsk, Poland). Then, the purified amplicons were subjected to Cycle Sequencing using BigDye™ Terminator v3.1 Cycle Sequencing Kit (Thermo Fisher Scientific, Waltham, MA, USA) following the manufacturer instruction concerning mix preparation and thermal protocol. Cycle sequencing products were purified using DyeEx 2.0 Spin Kit (Quiagen, Hilden, Germany). A total of 5 μL of DNA were loaded into a 96-well plate with 10 μL of formamide (Merck Millipore, Darmstadt, Germany) and analyzed by Abi Prism 310 Genetic Analyzer (Thermo Fisher Scientific, Waltham, MA, USA). A consensus sequence was generated using ClustalW [57], which was then compared with the data on NCBI (National Center for Biotechnology Information) using Nucleotide Blast (Blastn) [58] for species determination. Following identification, the obtained sequences were deposited on the GenBank database. 

### 2.4. Hsp65 Phylogenetic Tree 

As a further analysis for species determination, a phylogenetic tree was constructed using the data obtained by sequencing the *hsp65* gene fragment. Alignment and phylogenetic analysis were performed with a 421 bp fragment using MEGAX software [59]. The tree was built using sequences of reference strains of the various members of MFG, plus *M. chelonae* strain ATCC 35,749 with the function of outgroup. The statistical method used was maximum likelihood analysis with the general time reversible (GTR) nucleotide substitution model; 1000 bootstrap replications were performed.

## 3. Results

### 3.1. Strains Collection after Preliminary Identification

Following identification by biochemical tests, 130 strains of NTM belonging to MFG were isolated from diseased fish. All isolates were fast-growing (growth in less than seven days), non-chromogenic (no pigment production), alcohol-acid-resistant (ZN+) bacilli. The isolates were identified from 25 different fish species, mostly freshwater (110/130, *p* = 84.6%). Furthermore, the analyzed mycobacteria were mainly recovered from aquarium fish (109/130, *p* = 83.8%); the remaining microorganisms came from farmed fish species (21/130, *p* = 16.2%). All the strains collected were analyzed with biomolecular methods for the species determination. 

### 3.2. Sanger Sequencing and Species Determination

The DNA extracted from all the isolates was amplified by the method described in the previous section (see Material and Methods, Section 2.2). Agarose gel electrophoresis allowed to verify the correct size of all the amplicons. Then, all 130 stored strains were subjected to Sanger sequencing. The comparison of the sequences obtained with those deposited in the NCBI database allowed to identify all the mycobacteria as species belonging to the MFG, with an identity percentage between 98.34% and 100%. Specifically, the isolates belonged to 9 different species, divided as follows: 63 *M. peregrinum* (*p* = 48.5%), 38 *M. fortuitum* (*p* = 29.2%), 12 *M. senegalense* (*p* = 9.2%), four *M. arceuilense* (*p* = 3.1%), four *M. brisbanense* (*p* = 3.1%), four *M. conceptionense* (*p* = 3.1%), two *M. alvei* (*p* = 1.5%), two *M. setense* (*p* = 1.5%), and one *M. septicum* (*p* = 0.8%). Table 2 summarizes the results of the species identification, in relation to the fish species analyzed. Details of isolates, including GenBank Accession Number of the deposited sequences, are available in Supplementary Materials (Table S1). 

### 3.3. Hsp65 Phylogenetic Tree

**Figure 1:**
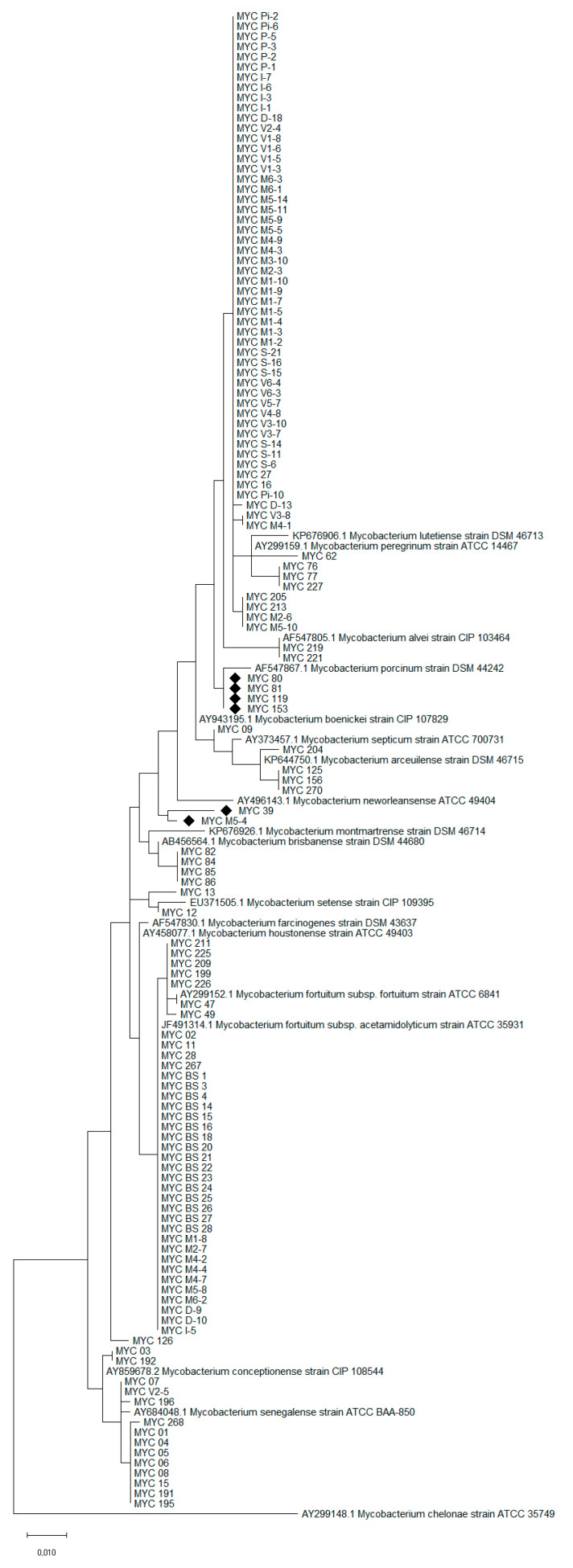
Phylogenetic tree obtained with a 421 bp fragment of the *hsp65* gene. The strains indicated with the symbol “♦” gave conflicting results between gene sequencing and phylogenetic analysis.

As a further analysis to determine the species of the isolates, a phylogenetic tree was construct with the obtained sequences compared with those of selected reference strains. The phylogenetic analysis substantially confirmed what previously obtained, except for 6 isolates (indicated by “♦” in the tree). The aforementioned strains are respectively MYC M5-4 (isolated from *Aulonocara* sp., identified as *M. fortuitum*, ID% 99.05), MYC 39 (isolated from *Colisa lalia*, identified as *M. conceptionense*, ID% 98.34), MYC 80 (isolated from *Acipenser ruthenus*, identified as *M. peregrinum*, ID% 99.76), MYC 81 (isolated from *Misgurnus* sp., identified as *M. peregrinum*, ID% 99.76), MYC 119 (isolated from *Hypostomus plecostomus*, identified as *M. peregrinum*, ID% 99.76), and MYC 153 (isolated from *Carassius auratus*, identified as *M. peregrinum*, ID% 99.76). Furthermore, with regard to the correctly-identified strains, the tree allowed to distinguish the *M. fortuitum* isolates in the two subspecies (Figure 1). 

## 4. Discussion

MFG members are globally distributed rapidly growing non-chromogenic mycobacteria. Since the first identification of the founding member of the group, *M. fortuitum*, the number of related species recognized has gradually increased in relation to the new identification techniques adopted. Given the wide distribution of these microorganisms, humans and animals can easily come into contact with them. In some cases, certain species of MFG are known to cause disease; among them, there are fish mycobacteriosis. Although *M. fortuitum* is one of the major etiological agents of these pathologies, the other MFG species are not to be excluded. In the present study, we tried to clarify the possible presence and role of the other species of MFG causing fish mycobacteriosis, in addition to *M. fortuitum*, using biomolecular techniques as an identification tool. 

In the first instance, 130 NTM strains preliminarily recognized with biochemical methods as MFG members were analyzed. These strains were isolated from fish that showed external and/or internal (granulomas) clinical signs. Analyzing the positive fish species and holding conditions, we have a fairly accurate representation of what is reported in the literature on fish mycobacteriosis. In fact, most of the species (109/130, *p* = 83.8%) come from aquaria, whose conditions (limited space, water recirculation, possible overcrowding) are predisposing factors for the onset mycobacteriosis in fish [49,60]. The remaining samples (21/130, *p* = 16.2%) came from farmed fish: Additoinally, in this case there are predisposing factors for the onset of infectious diseases, including high density in tanks and intensive farming conditions [61]. As further confirmation of the wide distribution of MFG species [15,62,63], the isolates come from both freshwater (110/130, *p* = 84.6%) and saltwater (20/130, *p* = 15.4%) fish. 

The preliminary identification of these isolates was carried out with biochemical tests. Among them, some are easy to perform and interpret (e.g., growth rate, pigment production, growth at different temperatures), given the presence of exhaustive data in the bibliography. For other tests, however, updated and necessary data are not always available in order to correctly given the result to determining the species of the isolate (see Table 1). In these cases, the data may lack or there may be a variable behavior between isolates of the same species (e.g., Tween-80 hydrolysis for *M. fortuitum*). It should also be considered that, although rapidly growing, the biochemical tests conducted on members of MFG are time consuming compared to the same ones performed on other more easily cultivable bacterial genera. 

In relation to this and to the increase in the number of recognized species [64,65], biomolecular methods are a quick, effective and cheaper way to NTM species determination. Our study appears to confirm this statement: In fact, all the isolates were identified at species level by *hsp65* gene sequencing with a percentage between 98.34 and 100%. Based on the study of McNabb and collaborators [66], the identification of species through sequence analysis of a portion of *hsp65* gene can be considered valid when the ID percentage is greater than 97%. Based on this, all identifications obtained were considered valid. The most isolated species was *M. peregrinum* (*p* = 48.5%). This species is already known as an etiological agent of fish mycobacteriosis, even if it is attributed a secondary role compared to other species (e.g., *M. marinum*, *M. chelonae*., *M. fortuitum*) [47]. Other authors have previously reported high prevalence of this species of mycobacterium [49]: It must therefore be considered whether these are sporadic cases linked to occasional mortality or whether there is an underestimation. To follow, the most identified species was *M. fortuitum* (*p* = 29.2%). Being among the species most involved in fish mycobacteriosis onset [47,48], our study confirms when already known from previous studies. As mentioned, the use of biomolecular techniques has led to the recognition of new species of MFG [14,64,67]. The application of these techniques in the diagnosis of fish mycobacteriosis has allowed to highlight nine different MFG species in our study. Previous studies using analytical methodologies comparable to those used in our study were able to identify these same species [52,53]. As several MFG species have also been found in humans [11,12,13,68], correct identification at species level is essential, without limiting to the use of methods such as biochemical tests. In fact, the improvement of diagnostic techniques is fundamental for these poor studied microorganisms to implement our knowledge, especially for their potential zoonotic implication. 

As a further test, a phylogenetic analysis was performed to confirm the sequencing results. Compare to gene sequencing, this analysis provides an easily interpretable and immediate result, even on a graphic level. Despite the high identification rates in sequencing, 6 isolates (*p* = 4.6%) did not confirm the results obtained following phylogenetic analysis. For example, MYC 80, MYC 81, MYC 119 and MYC 153 were all identified as *M. peregrinum* with an ID% of 99.76 (similarity with strain MK341520.1) following Blastn analysis; instead, the phylogenetic tree shows a greater resemblance to *M. porcinum* DSM 44242. These cases pose a known problem for those who work with gene sequences databases, that is the quality controls of the deposited sequences [69]. Finally, compared to Blastn identification, the phylogenetic tree allowed the division between the strains of *M. fortuitum* in the two related subspecies, further implementing the information regarding the identification of these isolates. 

## 5. Conclusions

NTM belonging to the MFG represent both a problem for fish and for public health, being potential zoonotic agents [47,52]. Being a group made up of several species, correct identification is the first step for accurate diagnosis and therapy, as well as to increase knowledge on relatively recent identified species (e.g., *M. arceuilense*, *M. lutetiense*, *M. montmartrense*). These little known and poorly diagnosed species pose several problems, including the use of adequate diagnostic techniques for the identification and assignment of a precise clinical significance. Our study confirmed that the use of a portion of the *hsp65* gene is a valid diagnostic tool for MFG species identification, through several hypervariable regions present in its sequence (Table 3). 

Despite the advantages, our study also highlighted several limitations in the methods used. First, the sequences present in the database must be of good quality for a correct identification of the species. Although qualitatively acceptable, some species have only single sequences for the *hsp65* gene. Therefore, although the *hsp65* gene provides more accurate identifications than other genes (e.g., rRNA16S) for MFG members, the most suitable approach in case of sequencing is the multigenic one [70]. Therefore, in cases of doubtful identification, the use of more fragments of the genome of these microorganisms is recommended, as a single gene able to discriminate all the species of the genus *Mycobacterium* is not yet known. In addition to sequencing, phylogenetic analysis provides a further diagnostic tool. Although there are advantages, even with respect to gene sequencing, phylogenetically similar species are a limitation for this method. In this perspective, a taxonomic revision of the MFG from a clinical point of view could bring advantages in the diagnostic process [71,72].

## Figures and Tables

**Table 1:** Cultural and biochemical characteristics of the MFG members. The information about the main cultural and biochemical tests used comes from Bergey’s Manual of Systematics Bacteriology, 2nd ed. [54], with additions on the species subsequently described [14].

	1	2	3	4	5	6	7	8	9	10	11	12	13	14	15	16	17
Growth at 22 °C	+	+	+	+	+	+	+	+	+	+	+	+	+	+	+	+	+
Growth at 28 °C	+	+	+	+	+	+	+	+	+	+	+	+	+	+	+	+	+
Growth at 37 °C	+	+	+	+	+	+	+	+	+	+	+	+	+	+	+	-	-
Growth at 42 °C	v	+	−	−	−	−	nd	+	−	−	+	nd	−	-	-	-	-
Growth at 45 °C	-	-	-	-	-	-	nd	nd	-	-	-	-	-	-	-	-	-
Growth on 5% NaCl	+	-	-	+	+	+	nd	+	+	+	+	+	+	+	nd	nd	nd
Growth on MacConkey Agar w/o crystal violet	+	+	-	+	+	nd	nd	+	+	+	nd	-	+	nd	nd	nd	nd
Pigment production	-	-	-	-	-	-	-	-	-	-	-	-	-	-	-	-	-
Nitrate reduction	+	+	+	+	+	+	+	+	+	+	-	+	+	+	+	-	v
Catalase at 22 °C	+	nd	+	+	nd	+	−	nd	nd	+	+	+	nd	nd	+	+	+
Catalase at 68 °C	+	nd	+	+	−	nd	nd	+	+	+	nd	nd	nd	+	+	v	−
Iron uptake	+	+	−		nd	+	−	nd	nd	+	nd	−	nd	+	−	−	−
Tween-80 hydrolysis	v	nd	+		nd	nd	nd	nd	nd	v	+	nd	nd	nd	+	+	+
Arylsulphatase (3 days)	+	+	+		+	+	nd	+	+	+	+	+	−	+	+	+	+
Arylsulphatase (7 days)	+	+	+		+	+	nd	+	+	+	+	+	nd	nd	nd	nd	nd
Urease	+	+	+		+	−	v	nd	+	+	+	+	nd	+	+	v	−
*Utilization of:*																	
Sodium citrate	−	−	−		−	−	+	−	−	−	+	+	nd	−	nd	nd	nd
Inositol	−	nd	−		nd	+	nd	nd	nd	+	+	nd	nd	nd	nd	nd	nd
Mannitol	v	−	−		nd	−	nd	nd	nd	nd	+	nd	nd	+	nd	nd	nd

Table interpretation. 1: *M. fortuitum* subsp. *fortuitum*; 2: *M. fortuitum* subsp. *acetamidolyticum*; 3: *M. alvei*; 4: *M. boenickei*; 5: *M. brisbanense*; 6: *M. conceptionense*; 7: *M. farcinogenes*; 8: *M. houstonense*: 9: *M. neworleansense*; 10: *M. peregrinum*; 11: *M. porcinum*; 12: *M. senegalense*; 13: *M. septicum*; 14: *M. setense*; 15: *M. arceuilense*; 16: *M. lutetiense*; 17: *M. montmartrense*; +: positive; −: negative; v: variable outcome; nd: not determined.

**Table 2:** Results of the identification by *hsp65* gene sequencing of the isolated strains.

Fish Species	1	2	3	4	5	6	7	8	9	10	11	12	13	14	15	16	Tot
*Acipenser ruthenus*	-	-	-	-	-	-	-	-	1	-	-	-	-	-	-	-	1
*Astatotilapia obliquidens*	1	-	-	-	-	-	-	-	-	-	-	-	-	-	-	-	1
*Aulonocara* sp.	2	-	-	-	-	-	-	-	6	-	-	-	-	-	-	-	8
*Botia macracantha*	-	-	-	-	-	-	-	-	-	-	1	-	-	-	-	-	1
*Capoeta tetrazona*	-	-	-	-	-	-	-	-	-	-	1	-	-	-	-	-	1
*Carassius auratus*	4	2	-	-	1	-	-	-	4	-	3	1	-	1	-	-	16
*Cyprinus carpio* var. *koi*	-	-	-	-	-	-	-	-	1	-	1	-	-	2	-	-	4
*Colisa lalia*	-	-	-	-	1	-	-	-	1	-	-	-	-	-	-	-	2
*Copadichromis borley*	3	-	-	-	-	-	-	-	3	-	-	-	-	-	-	-	6
*Copadichromis* sp.	2	-	-	-	-	-	-	-	13	-	-	-	-	-	-	-	15
*Dicentrarchus labrax*	16	-	-	-	-	-	-	-	-	-	-	-	-	-	-	-	16
*Garra rufa*	1	-	-	-	-	-	-	-	1	-	-	-	2	-	-	-	4
*Hypostomus plecostomus*	-	-	-	-	-	-	-	-	1	-	-	-	-	-	-	-	1
*Maylandia lombardoi*	-	-	-	-	-	-	-	-	4	-	-	-	-	-	-	-	4
*Misgurnus* sp.	-	-	-	-	-	-	-	-	1	-	-	-	-	-	-	-	1
*Nimbochromis livingstonii*	2	-	-	-	-	-	-	-	12	-	-	-	-	-	-	-	14
*Nimbochromis venustus*	1	-	-	-	-	-	-	-	3	-	-	-	-	-	-	-	4
*Placidochromis* sp.	1	-	-	-	-	-	-	-	7	-	1	-	-	-	-	-	9
*Poecilia latipinna*	1	-	-	-	-	-	-	-	-	-	-	-	-	1	-	-	2
*Poecilia reticulata*	3	-	-	-	-	-	-	-	-	-	-	-	-	-	-	-	3
*Pseudotropheus* sp.	-	-	-	-	-	-	-	-	3	-	-	-	-	-	-	-	3
*Pterophyllum scalare*	-	-	-	-	1	-	-	-	-	-	-	-	-	-	-	-	1
*Sciaenops ocellatus*	-	-	-	4	-	-	-	-	-	-	-	-	-	-	-	-	4
*Symphysodon discus*	1	-	-	-	1	-	-	-	-	-	5	-	-	-	-	-	7
*Xiphophorus maculatus*	-	-	-	-	-	-	-	-	2	-	-	-	-	-	-	-	2
	38	2	-	4	4	-	-	-	63	-	12	1	2	4	-	-	130

Table interpretation. 1: *M. fortuitum*; 2: *M. alvei*; 3: *M. boenickei*; 4: *M. brisbanense*; 5: *M. conceptionense*; 6: *M. farcinogenes*; 7: *M. houstonense*: 8: *M. neworleansense*; 9: *M. peregrinum*; 10: *M. porcinum*; 11: *M. senegalense*; 12: *M. septicum*; 13: *M. setense*; 14: *M. arceuilense*; 15: *M. lutetiense*; 16: *M. montmartrense*.

**Table 3:** Mutations in the *hsp65* gene sequences of the portion considered in the study found in the NCBI Nucleotide database. Reference strains indicated as type strain in Bergey’s manual [54] were considered. Nucleotide positions refer to those obtained by aligning type strains sequences with the complete genome of *M. fortuitum* subsp. *fortuitum* strain ATCC 6841 (Sequence ID: CP014258.1). The dot (·) indicates identical base compared to *M. fortuitum* subsp. *fortuitum* strain ATCC 6841 taken as reference species.

Nucleotide Position	1	2	3	4	5	6	7	8	9	10	11	12	13	14	15	16	17
5398016	T	·	·	·	·	·	·	·	·	·	·	·	C	·	·	·	·
5398010	C	·	G	G	G	G	·	·	·	G	G	G	G	G	G	G	G
5398004	A	·	·	·	·	G	·	·	·	·	·	G	·	·	·	·	·
5398001	G	·	·	·	·	A	·	·	·	·	·	A	·	·	·	·	·
5397986	C	·	·	·	·	T	·	·	T	·	·	T	T	·	T	·	·
5397976	G	·	·	·	·	·	·	·	·	·	T	·	·	·	·	·	·
5397974	G	·	·	·	·	·	·	·	·	·	·	·	·	A	·	·	·
5397971	C	·	·	·	·	T	·	·	·	T	T	T	·	·	·	·	·
5397968	C	·	·	·	·	·	·	·	·	·	·	·	·	·	·	T	·
5397941	A	·	T	C	C	C	C	C	C	C	C	C	C	C	C	C	T
5397935	C	·	·	·	·	·	·	·	·	·	·	·	·	·	A	·	·
5397926	T	·	C	C	C	·	C	C	C	C	C	·	C	C	C	C	C
5397920	T	·	·	·	C	·	·	·	·	·	·	·	·	·	·	C	·
5397914	C	·	·	·	·	·	·	·	·	·	·	T	·	·	·	·	·
5397890	G	·	·	·	·	·	·	·	·	·	·	·	·	·	·	·	T
5397860	C	·	T	·	·	·	·	·	·	T	·	·	·	·	·	T	T
5397857	C	·	·	·	·	·	·	·	G	·	·	·	·	G	·	·	·
5397854	G	·	·	·	·	·	·	·	A	·	·	·	·	A	·	·	·
5397839	G	·	C	C	·	·	·	·	C	C	C	·	C	·	C	C	·
5397836	G	·	C	C	·	·	·	·	C	C	T	·	C	·	C	C	·
5397833	G	·	·	·	·	C	C	·	·	·	·	C	·	·	·	·	·
5397829	A	·	T	T	·	·	·	·	T	T	T	·	T	·	T	T	·
5397828	G	·	C	C	·	·	·	·	C	C	C	·	C	·	C	C	·
5397797	C	·	·	·	·	·	·	·	·	·	T	·	·	·	·	·	·
5397783	G	·	·	·	·	·	·	·	·	·	·	C	·	·	·	·	·
5397782	T	·	·	·	·	·	·	·	·	·	·	G	·	·	·	·	·
5397776	C	·	·	·	·	·	·	·	·	·	·	G	·	·	·	·	·
5397773	C	·	·	·	·	·	·	·	·	·	·	G	·	·	·	·	·
5397770	T	·	·	A	A	·	·	·	·	A	A	C	A	·	A	A	A
5397766	C	·	·	·	·	·	·	·	·	·	·	A	·	·	·	·	·
5397765	A	·	·	·	·	·	·	·	·	·	·	C	·	·	·	·	·
5397764	G	·	·	·	·	·	·	·	·	·	·	C	·	·	·	·	·
5397763	T	·	·	·	·	·	·	·	·	·	·	C	·	·	·	·	·
5397762	C	·	·	·	·	·	·	·	·	·	·	A	·	·	·	·	·
5397761	C	·	·	·	·	·	·	·	·	·	·	G	·	·	·	·	·
5397755	T	·	C	C	·	·	·	·	·	C	C	C	C	C	C	C	C
5397754	G	·	·	·	·	·	·	·	·	·	·	A	·	·	·	·	·
5397752	C	·	·	·	·	·	·	·	·	·	·	G	·	·	·	·	·
5397701	C	·	·	·	·	·	·	·	·	·	·	T	·	·	·	·	·
5397694	A	·	T	·	·	·	·	·	·	·	·	T	·	·	·	·	·
5397693	G	·	C	·	·	·	·	·	·	·	·	C	·	·	·	·	·
5397692	C	·	G	·	·	·	·	·	·	·	·	·	·	·	·	·	·
5397680	C	·	G	·	G	·	·	·	·	G	G	G	T	·	G	G	G
5397677	G	·	C	C	·	·	·	·	·	·	C	·	C	·	·	C	·
5397671	G	·	·	·	·	·	·	·	·	·	·	C	·	C	·	·	·
5397656	T	·	·	·	·	·	·	·	·	C	·	·	C	·	C	C	C
5397650	C	G	G	G	G	G	G	G	G	·	·	·	·	·	·	·	·

Table interpretation. 1: *M. fortuitum* subsp. *fortuitum* ATCC 6841; 2: *M. fortuitum* subsp. *acetamidolyticum* ATCC 35931; 3: *M. alvei* CIP 103464; 4: *M. boenickei* CIP 107829; 5: *M. brisbanense* DSM 44680; 6: *M. conceptionense* CIP 108544; 7: *M. farcinogenes* DSM 43637; 8: *M. houstonense* ATCC 49403: 9: *M. neworleansense* ATCC 49404; 10: *M. peregrinum* ATCC 14467; 11: *M. porcinum* DSM 44242; 12: *M. senegalense* ATCC 35796; 13: *M. septicum* ATCC 700731; 14: *M. setense* CIP 109395; 15: *M. arceuilense* DSM 46715; 16: *M. lutetiense* DSM 46713; 17: *M. montmartrense* DSM 46714.

## Data Availability

Not applicable.

## Supplementary Materials

The following are available online at https://www.mdpi.com/article/10.3390/microorganisms9040797/s1, Table S1. Details about the strains analyzed in the study.
