# Supplementary material for: Gene Sequencing and Phylogenetic Analysis: Powerful Tools for an Improved Diagnosis of Fish Mycobacteriosis Caused by Mycobacterium fortuitum Group Members
Source: Microorganisms. 2021 Apr 10;9(4):797. doi: 10.3390/microorganisms9040797 (PMC8068823; doi:10.3390/microorganisms9040797)
Supplement: Supplementary file 1 [file microorganisms-09-00797-s001.pdf]

microorganisms

Article

Gene Sequencing and Phylogenetic Analysis: Powerful Tools for an Improved Diagnosis of Fish Mycobacteriosis Caused by *Mycobacterium fortuitum* Group Members

Davide Mugetti [1,*], Mattia Tomasoni [1], Paolo Pastorino [1], Giuseppe Esposito [2], Vasco Menconi [1], Alessandro Dondo [1] and Marino Prearo [1]

[1] Istituto Zooprofilattico Sperimentale del Piemonte, Liguria e Valle d'Aosta, Via Bologna 148, 10154 Torino, Italy; mattia.tomasoni@izsto.it (M.T.); paolo.pastorino@izsto.it (P.P.); vasco.menconi@izsto.it (V.M.); alessandro.dondo@izsto.it (A.D.); marino.prearo@izsto.it (M.P.)
[2] Dipartimento di Medicina Veterinaria, Università degli Studi di Sassari, Via Vienna 2, 07100 Sassari, Italy; gsesposito@uniss.it (G.E.)
* Correspondence: davide.mugetti@izsto.it; Tel.: +39-0112686251

Table S1. Details about the strains analyzed in the study. The GenBank Accession Number is the same for isolates with all identical information (year of isolation, infected fish species, isolate identification and ID %).

Year of isolation	Isolate	Infected fish species	Isolate identification	ID %	GenBank Accession Number
2015	MYC BS 1	*Dicentrarchus labrax*	*M. fortuitum*	100	MW731514
2015	MYC BS 3	*Dicentrarchus labrax*	*M. fortuitum*	100	MW731514
2015	MYC BS 4	*Dicentrarchus labrax*	*M. fortuitum*	100	MW731514
2015	MYC BS 14	*Dicentrarchus labrax*	*M. fortuitum*	100	MW731514
2015	MYC BS 15	*Dicentrarchus labrax*	*M. fortuitum*	100	MW731514
2015	MYC BS 16	*Dicentrarchus labrax*	*M. fortuitum*	100	MW731514
2015	MYC BS 18	*Dicentrarchus labrax*	*M. fortuitum*	100	MW731514
2015	MYC BS 20	*Dicentrarchus labrax*	*M. fortuitum*	100	MW731514
2015	MYC BS 21	*Dicentrarchus labrax*	*M. fortuitum*	100	MW731514
2015	MYC BS 22	*Dicentrarchus labrax*	*M. fortuitum*	100	MW731514
2015	MYC BS 23	*Dicentrarchus labrax*	*M. fortuitum*	100	MW731514
2015	MYC BS 24	*Dicentrarchus labrax*	*M. fortuitum*	100	MW731514
2015	MYC BS 25	*Dicentrarchus labrax*	*M. fortuitum*	100	MW731514
2015	MYC BS 26	*Dicentrarchus labrax*	*M. fortuitum*	100	MW731514
2015	MYC BS 27	*Dicentrarchus labrax*	*M. fortuitum*	100	MW731514
2015	MYC BS 28	*Dicentrarchus labrax*	*M. fortuitum*	100	MW731514
2015	MYC 7	*Symphysodon discus*	*M. senegalense*	100	MW731480
2015	MYC 12	*Garra rufa*	*M. setense*	100	MW731484
2015	MYC 13	*Garra rufa*	*M. setense*	99	MW731485
2015	MYC 15	*Capoeta tetrazona*	*M. senegalense*	100	MW731486
2015	MYC 16	*Carassius auratus*	*M. peregrinum*	100	MW731487
2015	MYC 27	*Garra rufa*	*M. peregrinum*	100	MW731488
2015	MYC 28	*Garra rufa*	*M. fortuitum*	100	MW731489
2017	MYC D-9	*Copadichromis borley*	*M. fortuitum*	100	MW731531
2017	MYC D-10	*Copadichromis borley*	*M. fortuitum*	100	MW731531
2017	MYC D-13	*Nimbochromis livingstonii*	*M. peregrinum*	99,76	MW731532
2017	MYC D-18	*Copadichromis* sp.	*M. peregrinum*	100	MW731533
2017	MYC I-1	*Nimbochromis livingstonii*	*M. peregrinum*	100	MW731534

**Year of isolation**	**Isolate**	**Infected fish species**	**Isolate identification**	**ID %**	**GenBank Accession Number**
2017	MYC I-3	*Copadichromis* sp.	*M. peregrinum*	100	MW731533
2017	MYC I-5	*Copadichromis borley*	*M. fortuitum*	100	MW731531
2017	MYC I-6	*Copadichromis* sp.	*M. peregrinum*	100	MW731533
2017	MYC I-7	*Copadichromis borley*	*M. peregrinum*	100	MW731535
2017	MYC M1-2	*Aulonocara* sp.	*M. peregrinum*	100	MW731516
2017	MYC M1-3	*Maylandia lombardoi*	*M. peregrinum*	100	MW731517
2017	MYC M1-4	*Copadichromis borley*	*M. peregrinum*	100	MW731535
2017	MYC M1-5	*Copadichromis* sp.	*M. peregrinum*	100	MW731533
2017	MYC M1-7	*Nimbochromis livingstonii*	*M. peregrinum*	100	MW731534
2017	MYC M1-8	*Copadichromis* sp.	*M. fortuitum*	100	MW731518
2017	MYC M1-9	*Placidochromis* sp.	*M. peregrinum*	100	MW731519
2017	MYC M1-10	*Copadichromis* sp.	*M. peregrinum*	100	MW731533
2017	MYC M2-3	*Pseudotropheus* sp.	*M. peregrinum*	100	MW731520
2017	MYC M2-6	*Copadichromis borley*	*M. peregrinum*	99,76	MW731521
2017	MYC M2-7	*Placidochromis* sp.	*M. fortuitum*	100	MW731522
2017	MYC M3-10	*Aulonocara* sp.	*M. peregrinum*	100	MW731516
2017	MYC M4-1	*Placidochromis* sp.	*M. peregrinum*	99,76	MW731523
2017	MYC M4-2	*Nimbochromis livingstonii*	*M. fortuitum*	100	MW731524
2017	MYC M4-3	*Copadichromis* sp.	*M. peregrinum*	100	MW731533
2017	MYC M4-4	*Aulonocara* sp.	*M. fortuitum*	100	MW731525
2017	MYC M4-7	*Copadichromis* sp.	*M. fortuitum*	100	MW731518
2017	MYC M4-9	*Copadichromis* sp.	*M. peregrinum*	100	MW731533
2017	MYC M5-4	*Aulonocara* sp.	*M. fortuitum*	99,05	MW731526
2017	MYC M5-5	*Maylandia lombardoi*	*M. peregrinum*	100	MW731517
2017	MYC M5-8	*Nimbochromis livingstonii*	*M. fortuitum*	100	MW731524
2017	MYC M5-9	*Pseudotropheus* sp.	*M. peregrinum*	100	MW731520
2017	MYC M5-10	*Nimbochromis livingstonii*	*M. peregrinum*	99,76	MW731527
2017	MYC M5-11	*Nimbochromis venustus*	*M. peregrinum*	100	MW731528
2017	MYC M5-14	*Nimbochromis livingstonii*	*M. peregrinum*	100	MW731534
2017	MYC M6-1	*Placidochromis* sp.	*M. peregrinum*	100	MW731519
2017	MYC M6-2	*Nimbochromis venustus*	*M. fortuitum*	100	MW731529
2017	MYC M6-3	*Nimbochromis livingstonii*	*M. peregrinum*	100	MW731534
2017	MYC 1	*Symphysodon discus*	*M. senegalense*	100	MW731477
2017	MYC 2	*Symphysodon discus*	*M. fortuitum*	100	MW731478
2017	MYC 3	*Symphysodon discus*	*M. conceptionense*	99,76	MW731479
2017	MYC 4	*Symphysodon discus*	*M. senegalense*	100	MW731477
2017	MYC 5	*Symphysodon discus*	*M. senegalense*	100	MW731477
2017	MYC 6	*Symphysodon discus*	*M. senegalense*	100	MW731477
2017	MYC 8	*Cyprinus carpio* var. *koi*	*M. senegalense*	100	MW731481
2017	MYC 9	*Carassius auratus*	*M. septicum*	99,76	MW731482
2017	MYC Pi-2	*Placidochromis* sp.	*M. peregrinum*	100	MW731519
2017	MYC Pi-6	*Nimbochromis venustus*	*M. peregrinum*	100	MW731528
2017	MYC Pi-10	*Copadichromis* sp.	*M. peregrinum*	100	MW731533
2017	MYC P-1	*Placidochromis* sp.	*M. peregrinum*	100	MW731519
2017	MYC P-2	*Nimbochromis livingstonii*	*M. peregrinum*	100	MW731534
2017	MYC P-3	*Nimbochromis livingstonii*	*M. peregrinum*	100	MW731534
2017	MYC P-5	*Nimbochromis livingstonii*	*M. peregrinum*	100	MW731534
2017	MYC S-6	*Aulonocara* sp.	*M. peregrinum*	100	MW731516
2017	MYC S-11	*Nimbochromis livingstonii*	*M. peregrinum*	100	MW731534

Year of isolation	Isolate	Infected fish species	Isolate identification	ID %	GenBank Accession Number
2017	MYC S-14	*Nimbochromis livingstonii*	*M. peregrinum*	100	MW731534
2017	MYC S-15	*Aulonocara* sp.	*M. peregrinum*	100	MW731516
2017	MYC S16	*Nimbochromis livingstonii*	*M. peregrinum*	100	MW731534
2017	MYC S-21	*Copadichromis* sp.	*M. peregrinum*	100	MW731533
2017	MYC V1-3	*Maylandia lombardoi*	*M. peregrinum*	100	MW731517
2017	MYC V1-5	*Nimbochromis venustus*	*M. peregrinum*	100	MW731528
2017	MYC V1-6	*Pseudotropheus* sp.	*M. peregrinum*	100	MW731520
2017	MYC V1-8	*Maylandia lombardoi*	*M. peregrinum*	100	MW731517
2017	MYC V2-4	*Placidochromis* sp.	*M. peregrinum*	100	MW731519
2017	MYC V2-5	*Placidochromis* sp.	*M. senegalense*	100	MW731530
2017	MYC V3-7	*Aulonocara* sp.	*M. peregrinum*	100	MW731516
2017	MYC V3-8	*Copadichromis* sp.	*M. peregrinum*	99,76	MW731515
2017	MYC V3-10	*Copadichromis* sp.	*M. peregrinum*	100	MW731533
2017	MYC V4-8	*Aulonocara* sp.	*M. peregrinum*	100	MW731516
2017	MYC V5-7	*Copadichromis* sp.	*M. peregrinum*	100	MW731533
2017	MYC V6-3	*Copadichromis* sp.	*M. peregrinum*	100	MW731533
2017	MYC V6-4	*Placidochromis* sp.	*M. peregrinum*	100	MW731519
2018	MYC 11	*Astatotilapia obliquidens*	*M. fortuitum*	100	MW731483
2018	MYC 39	*Colisa lalia*	*M. conceptionense*	98,34	MW731490
2018	MYC 47	*Poecilia latipinna*	*M. fortuitum*	99,76	MW731491
2018	MYC 49	*Carassius auratus*	*M. fortuitum*	99,76	MW731492
2018	MYC 62	*Cyprinus carpio* var. *koi*	*M. peregrinum*	98,34	MW731493
2018	MYC 76	*Xiphophorus maculatus*	*M. peregrinum*	98,81	MW731494
2018	MYC 77	*Xiphophorus maculatus*	*M. peregrinum*	98,81	MW731494
2018	MYC 80	*Acipenser ruthenus*	*M. peregrinum*	99,76	MW731495
2018	MYC 81	*Misgurnus* sp.	*M. peregrinum*	99,76	MW731496
2018	MYC 82	*Sciaenops ocellatus*	*M. brisbanense*	99,51	MW731497
2018	MYC 84	*Sciaenops ocellatus*	*M. brisbanense*	99,51	MW731497
2018	MYC 85	*Sciaenops ocellatus*	*M. brisbanense*	99,51	MW731497
2018	MYC 86	*Sciaenops ocellatus*	*M. brisbanense*	99,51	MW731497
2018	MYC 119	*Hypostomus plecostomus*	*M. peregrinum*	99,76	MW731498
2018	MYC 125	*Poecilia latipinna*	*M. arceuilense*	99,52	MW731499
2019	MYC 126	*Pterophyllum scalare*	*M. conceptionense*	98,57	MW731500
2019	MYC 153	*Carassius auratus*	*M. peregrinum*	99,76	MW731501
2019	MYC 156	*Cyprinus carpio* var. *koi*	*M. arceuilense*	99,52	MW731502
2019	MYC 191	*Carassius auratus*	*M. senegalense*	100	MW731503
2019	MYC 192	*Carassius auratus*	*M. conceptionense*	99,76	MW731504
2019	MYC 195	*Carassius auratus*	*M. senegalense*	100	MW731503
2019	MYC 196	*Botia macracantha*	*M. senegalense*	99,76	MW731505
2019	MYC 199	*Poecilia reticulata*	*M. fortuitum*	100	MW731506
2019	MYC 204	*Carassius auratus*	*M. arceuilense*	99,52	MW731507
2019	MYC 205	*Carassius auratus*	*M. peregrinum*	99,76	MW731508
2019	MYC 209	*Poecilia reticulata*	*M. fortuitum*	100	MW731506
2019	MYC 211	*Poecilia reticulata*	*M. fortuitum*	100	MW731506
2019	MYC 213	*Carassius auratus*	*M. peregrinum*	99,76	MW731508
2019	MYC 219	*Carassius auratus*	*M. alvei*	99,52	MW731509
2019	MYC 221	*Carassius auratus*	*M. alvei*	99,52	MW731509
2019	MYC 225	*Carassius auratus*	*M. fortuitum*	100	MW731510
2019	MYC 226	*Carassius auratus*	*M. fortuitum*	100	MW731510

Year of isolation	Isolate	Infected fish species	Isolate identification	ID %	GenBank Accession Number
2019	MYC 227	*Colisa lalia*	*M. peregrinum*	98,81	MW731511
2019	MYC 267	*Carassius auratus*	*M. fortuitum*	100	MW731512
2019	MYC 268	*Carassius auratus*	*M. senegalense*	99,76	MW731513
2019	MYC 270	*Cyprinus carpio* var. *koi*	*M. arceuilense*	99,52	MW731502
